# Supplementary material for: Hybrid quantum search with genetic algorithm optimization
Source: PeerJ Comput Sci. 2024 Aug 5;10:e2210. doi: 10.7717/peerj-cs.2210 (PMC11639129; doi:10.7717/peerj-cs.2210)
Supplement: Supplemental Information 1 [file peerj-cs-10-2210-s001.pdf]

Hybrid quantum search with genetic algorithm optimization

(Supplementary Information)

Sebastian Mihai Ardelean, Mihai Udrescu

1 Additional experimental results

1.1 Knapsack problem

1.1.1 Conventional GA with non-adaptive mutation

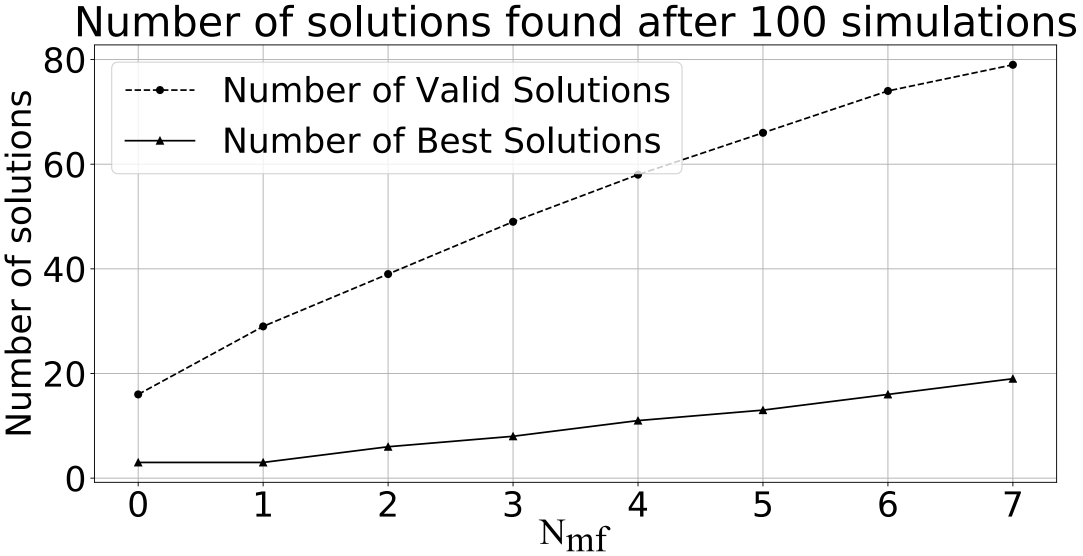

Figure S1: The number of valid and best solutions found by pure quantum Hybrid Quantum Algorithm with Genetic Optimization (HQAGO) (0 fixed qubits) after running the algorithm 100 times. The best outcome, in terms of the best and valid solutions, is achieved after 8 RQGA generations.

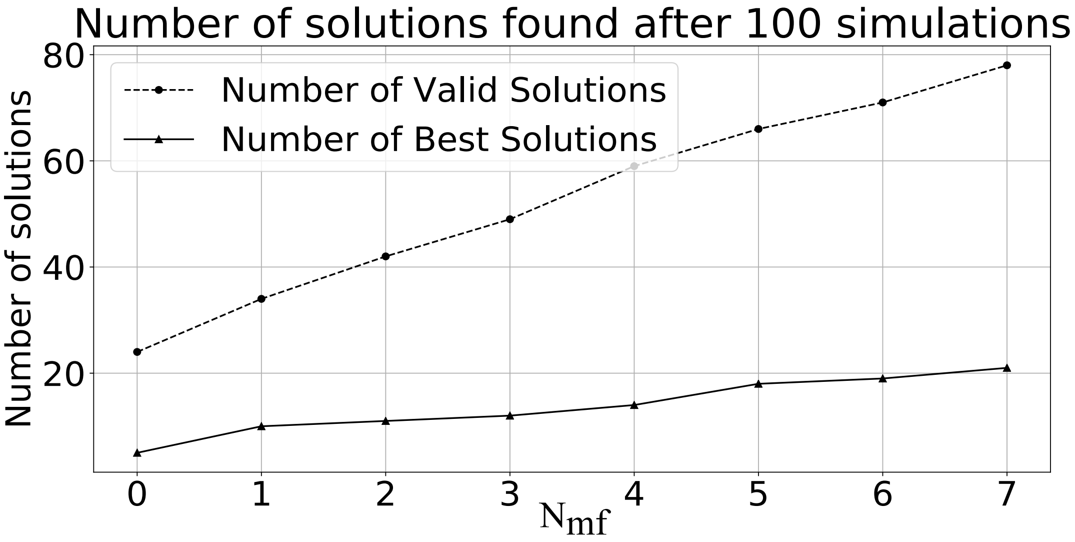

Figure S2: The number of valid and best solutions found by Hybrid Quantum Algorithm with Genetic Optimization (HQAGO) with 1 fixed qubit after running the algorithm 100 times. The best outcome, in terms of the best and valid solutions, is achieved after 8 RQGA generations.

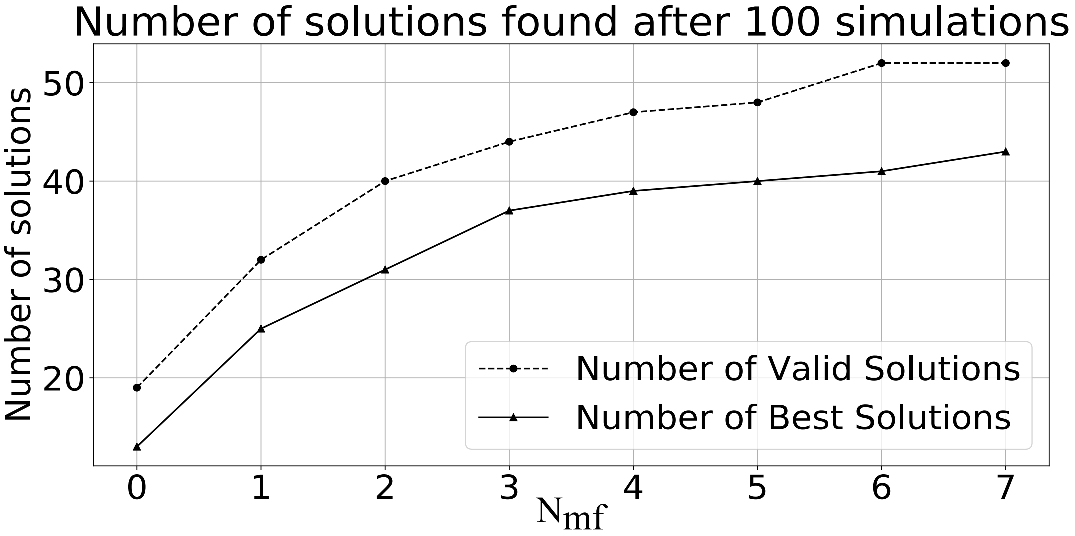

Figure S3: The number of valid and best solutions found by Hybrid Quantum Algorithm with Genetic Optimization (HQAGO) with 2 fixed qubits after running the algorithm 100 times. The best outcome, in terms of the best and valid solutions, is achieved after 8 RQGA generations.

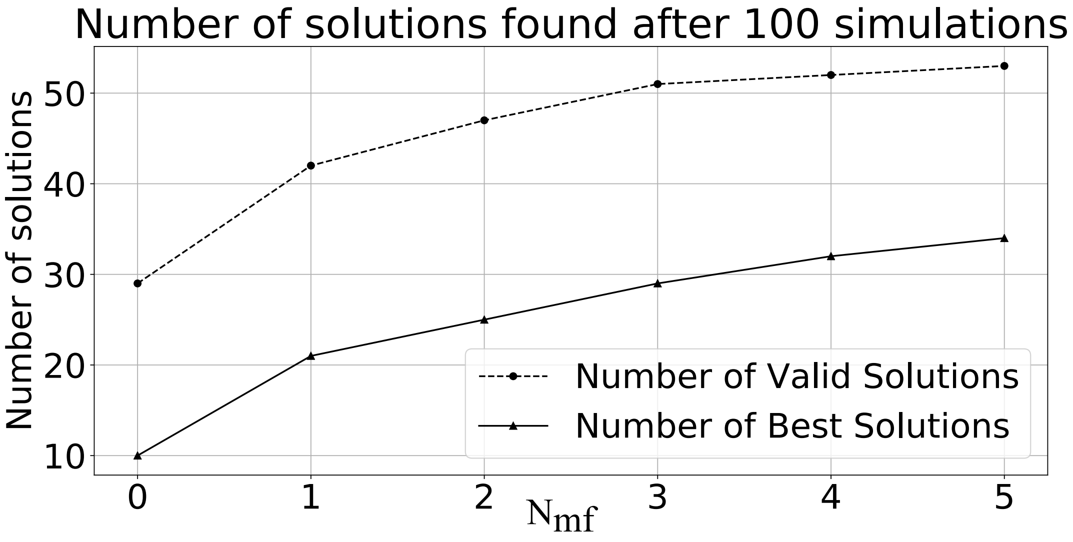

Figure S4: The number of valid and best solutions found by Hybrid Quantum Algorithm with Genetic Optimization (HQAGO) with 3 fixed qubits after running the algorithm 100 times. The best outcome, in terms of the best and valid solutions, is achieved after 6 RQGA generations.

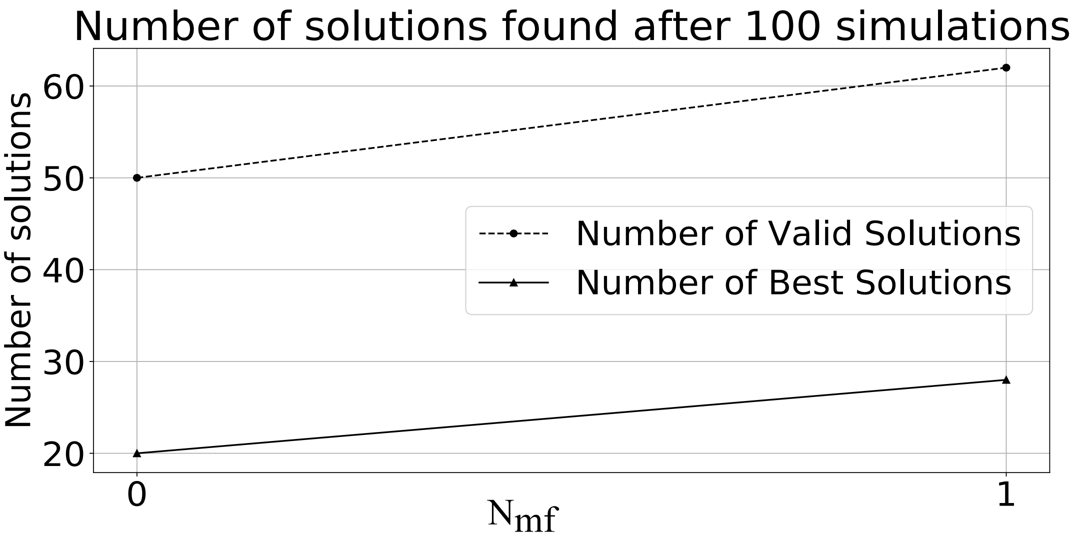

Figure S5: The number of valid and best solutions found by Hybrid Quantum Algorithm with Genetic Optimization (HQAGO) with 4 fixed qubits after running the algorithm 100 times. The best outcome, in terms of the best and valid solutions, is achieved after 2 RQGA generation.

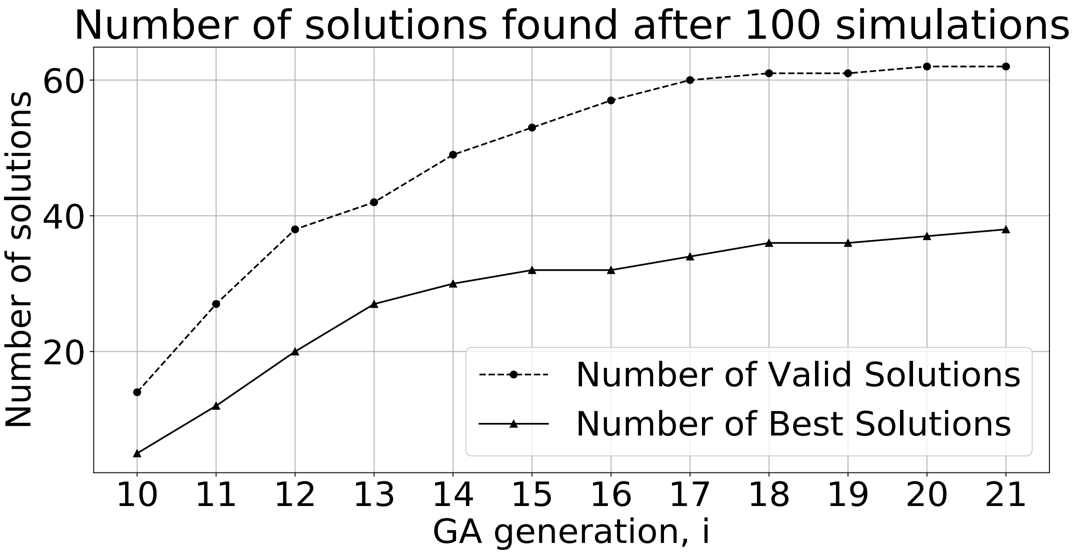

Figure S6: The number of valid and best solutions found by Hybrid Quantum Algorithm with Genetic Optimization (HQAGO) with 5 fixed qubits (representing a classic GA) after running the algorithm 100 times. The best outcome, in terms of the best and valid solutions, is achieved after 21 GA generations.

1.1.2 Conventional GA with adaptive mutation probabilities

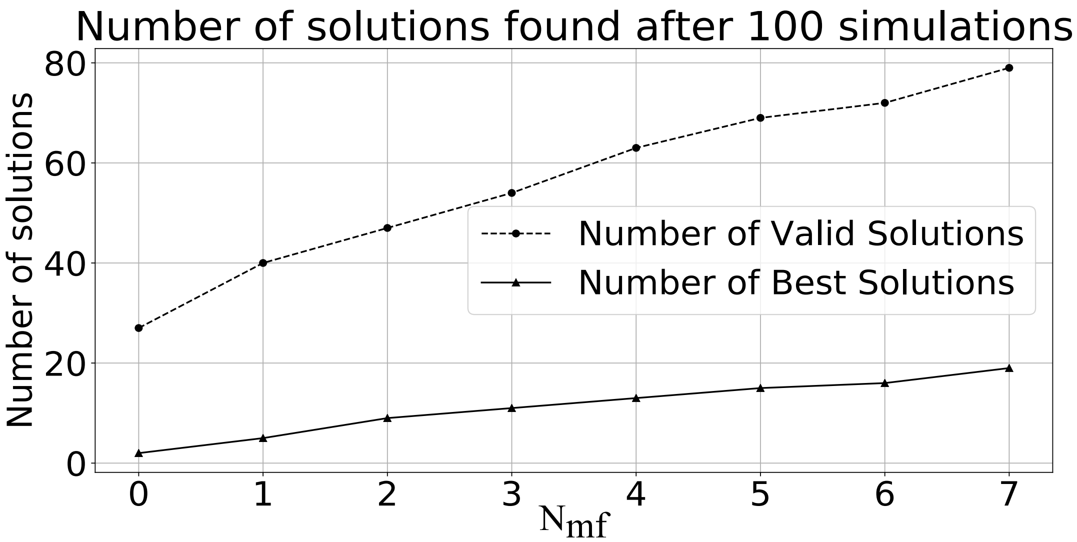

Figure S7: The number of valid and best solutions found by pure quantum Hybrid Quantum Algorithm with Genetic Optimization (HQAGO) (0 fixed qubits) after running the algorithm 100 times. The best outcome, in terms of the best and valid solutions, is achieved after 8 RQGA generations.

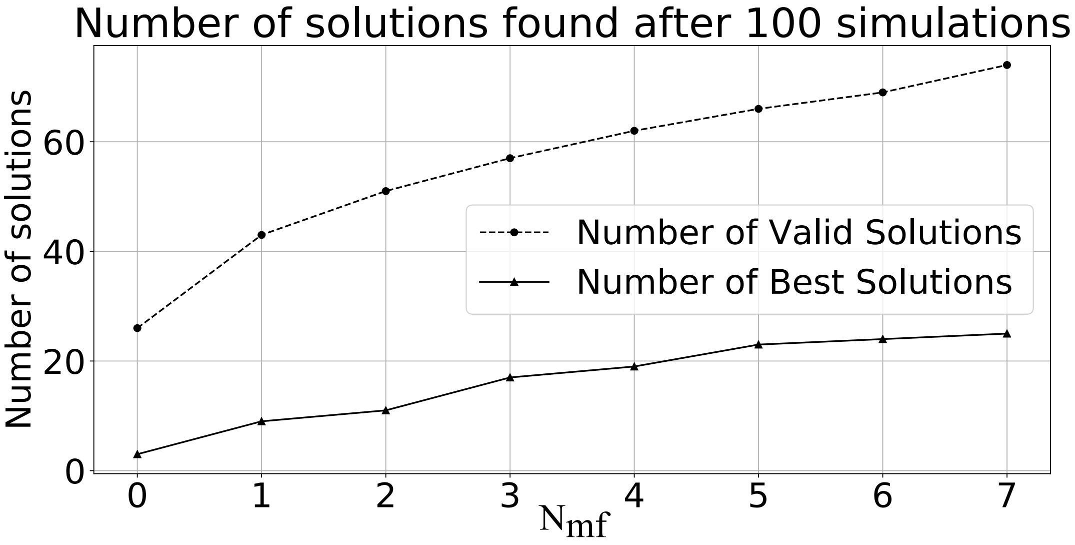

Figure S8: The number of valid and best solutions found by Hybrid Quantum Algorithm with Genetic Optimization (HQAGO) with 1 fixed qubit after running the algorithm 100 times. The best outcome, in terms of the best and valid solutions, is achieved after 8 RQGA generations.

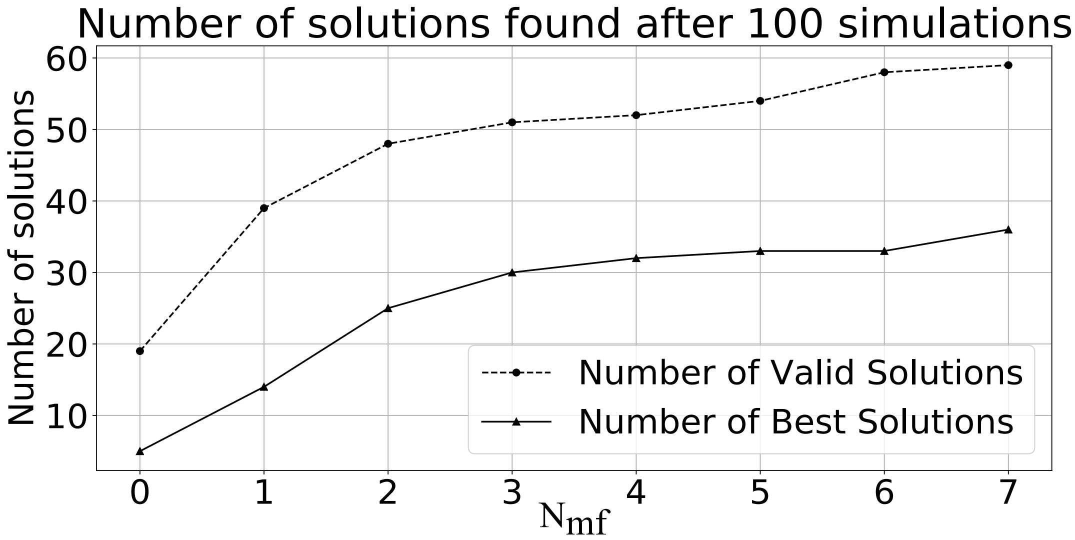

Figure S9: The number of valid and best solutions found by Hybrid Quantum Algorithm with Genetic Optimization (HQAGO) with 2 fixed qubits after running the algorithm 100 times. The best outcome, in terms of the best and valid solutions, is achieved after 8 RQGA generations.

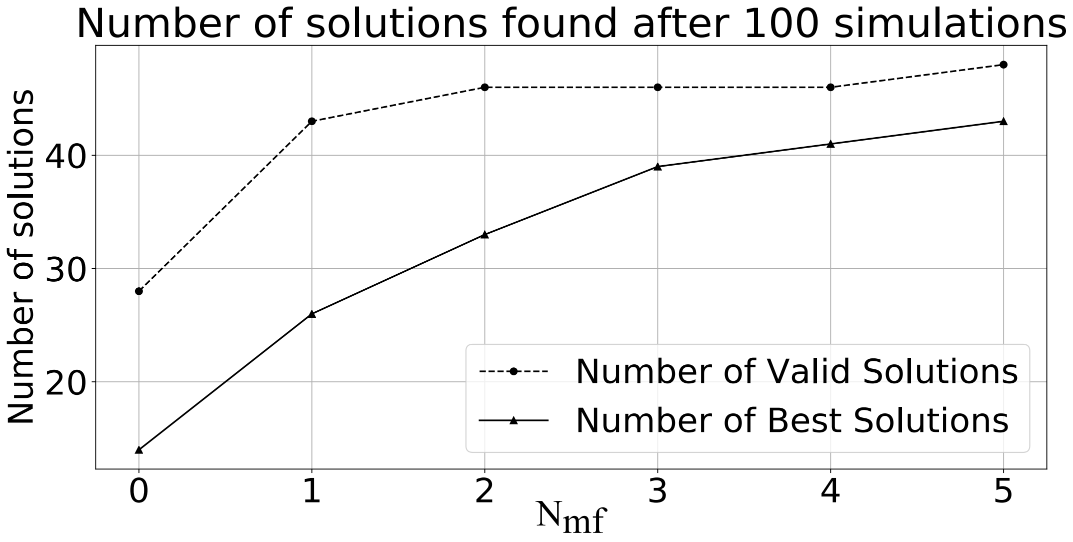

Figure S10: The number of valid and best solutions found by Hybrid Quantum Algorithm with Genetic Optimization (HQAGO) with 3 fixed qubits after running the algorithm 100 times. The best outcome, in terms of the best and valid solutions, is achieved after 6 RQGA generations.

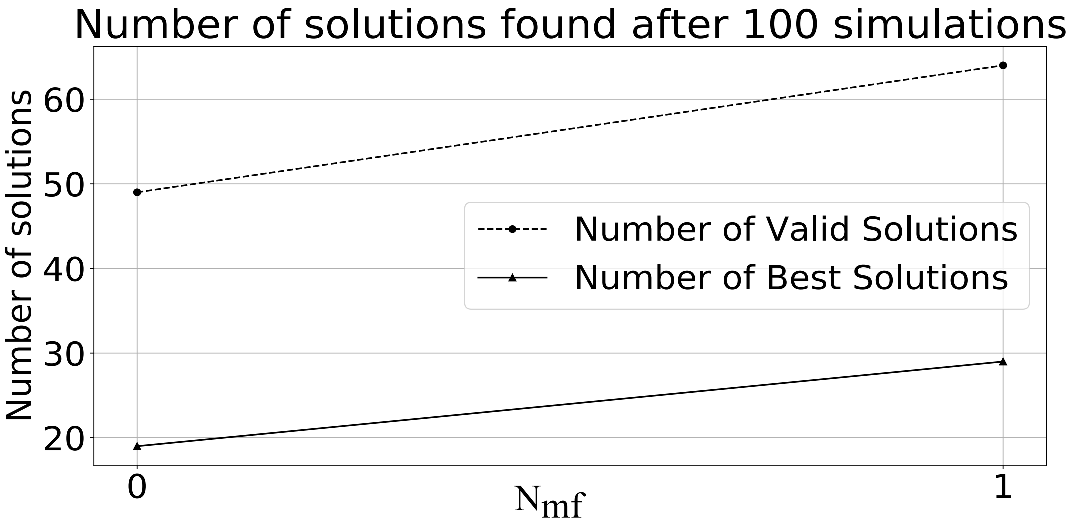

Figure S11: The number of valid and best solutions found by Hybrid Quantum Algorithm with Genetic Optimization (HQAGO) with 4 fixed qubits after running the algorithm 100 times. The best outcome, in terms of the best and valid solutions, is achieved after 2 RQGA generation.

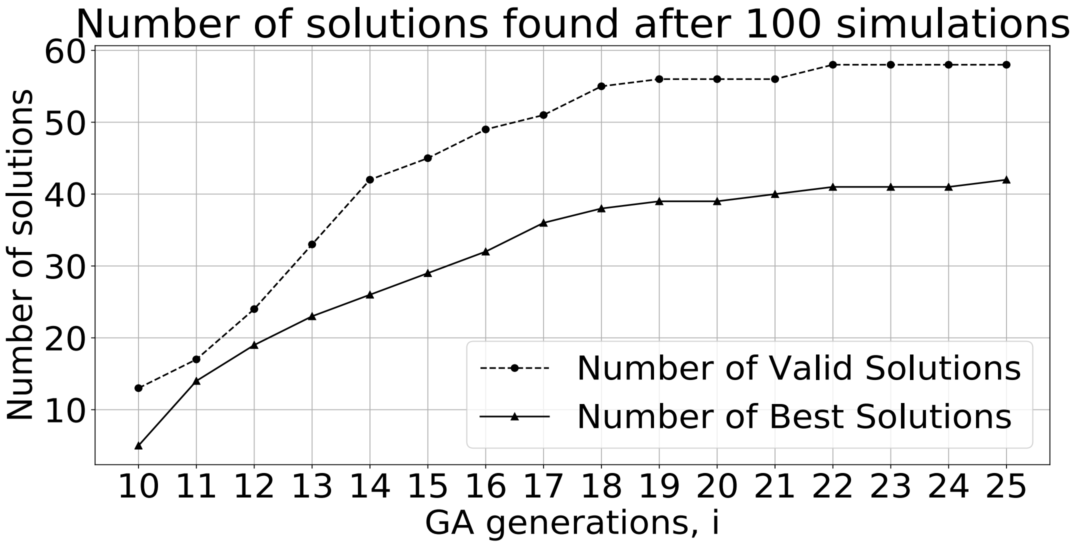

Figure S12: The number of valid and best solutions found by Hybrid Quantum Algorithm with Genetic Optimization (HQAGO) with 5 fixed qubits (representing a classic GA) after running the algorithm 100 times. The best outcome, in terms of the best and valid solutions, is achieved after 22 GA generations.

1.1.3 Conventional GA with adaptive percentage of the mutated genes

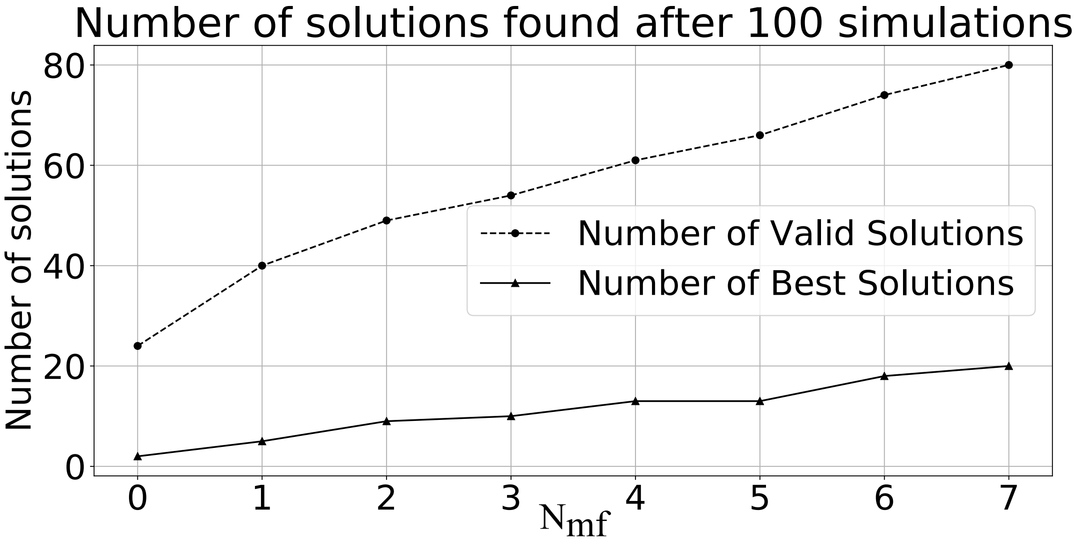

Figure S13: The number of valid and best solutions found by pure quantum Hybrid Quantum Algorithm with Genetic Optimization (HQAGO) (0 fixed qubits) after running the algorithm 100 times. The best outcome, in terms of the best and valid solutions, is achieved after 8 RQGA generations.

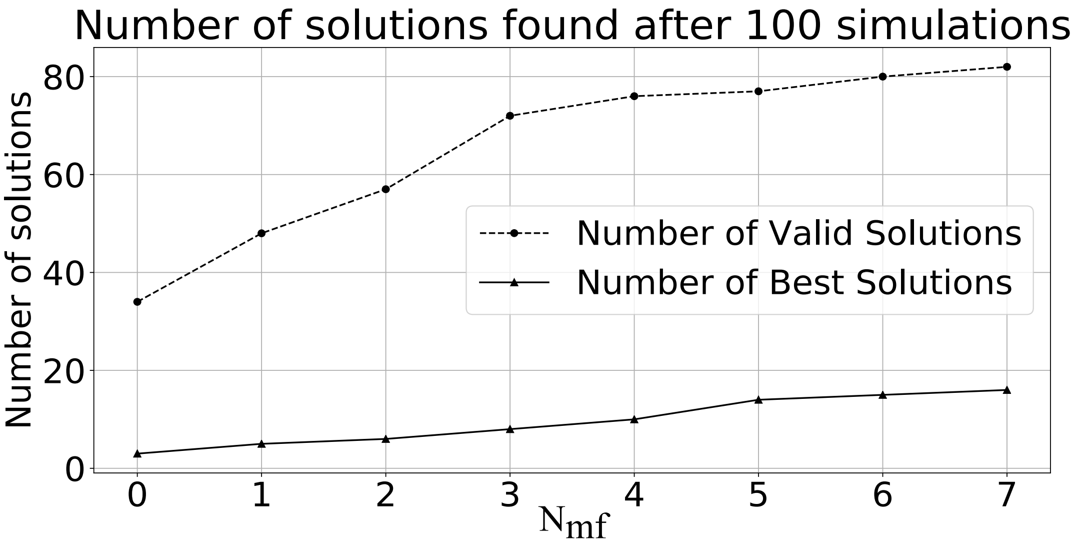

Figure S14: The number of valid and best solutions found by Hybrid Quantum Algorithm with Genetic Optimization (HQAGO) with 1 fixed qubit after running the algorithm 100 times. The best outcome, in terms of the best and valid solutions, is achieved after 8 RQGA generations.

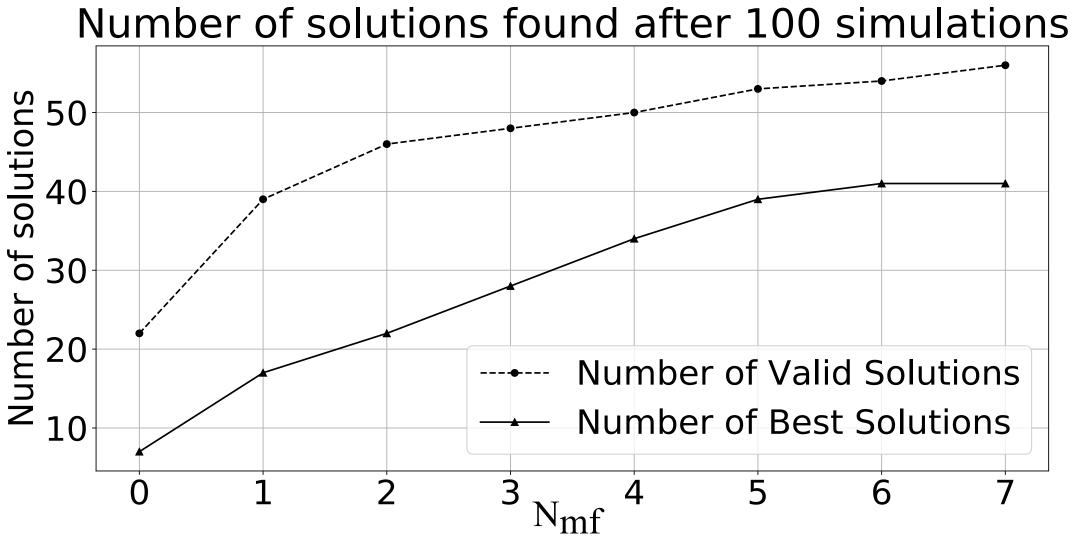

Figure S15: The number of valid and best solutions found by Hybrid Quantum Algorithm with Genetic Optimization (HQAGO) with 2 fixed qubits after running the algorithm 100 times. The best outcome, in terms of the best and valid solutions, is achieved after 8 RQGA generations.

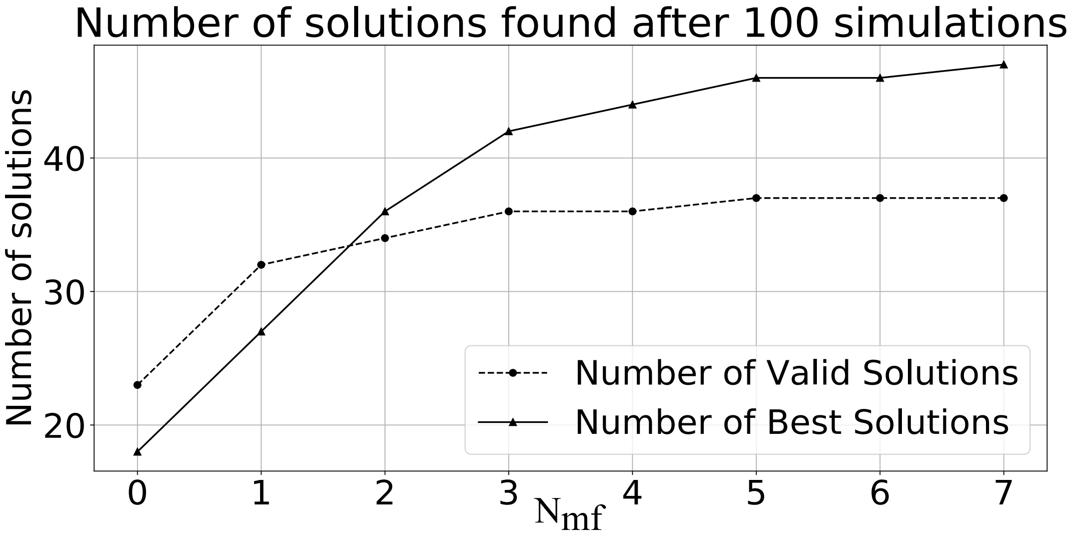

Figure S16: The number of valid and best solutions found by Hybrid Quantum Algorithm with Genetic Optimization (HQAGO) with 3 fixed qubits after running the algorithm 100 times. The best outcome, in terms of the best and valid solutions, is achieved after 6 RQGA generations.

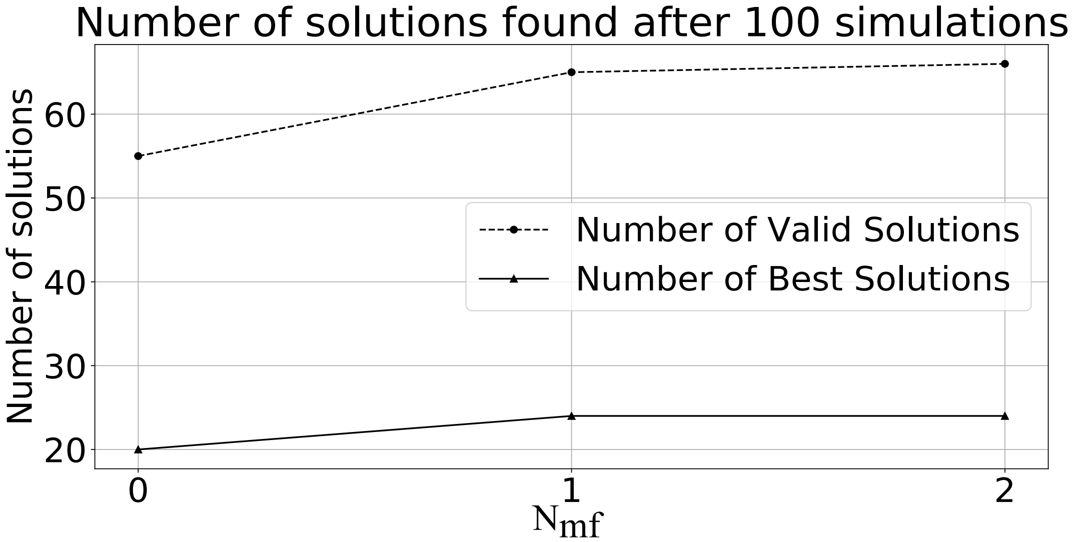

Figure S17: The number of valid and best solutions found by Hybrid Quantum Algorithm with Genetic Optimization (HQAGO) with 4 fixed qubits after running the algorithm 100 times. The best outcome, in terms of the best and valid solutions, is achieved after 2 RQGA generation.

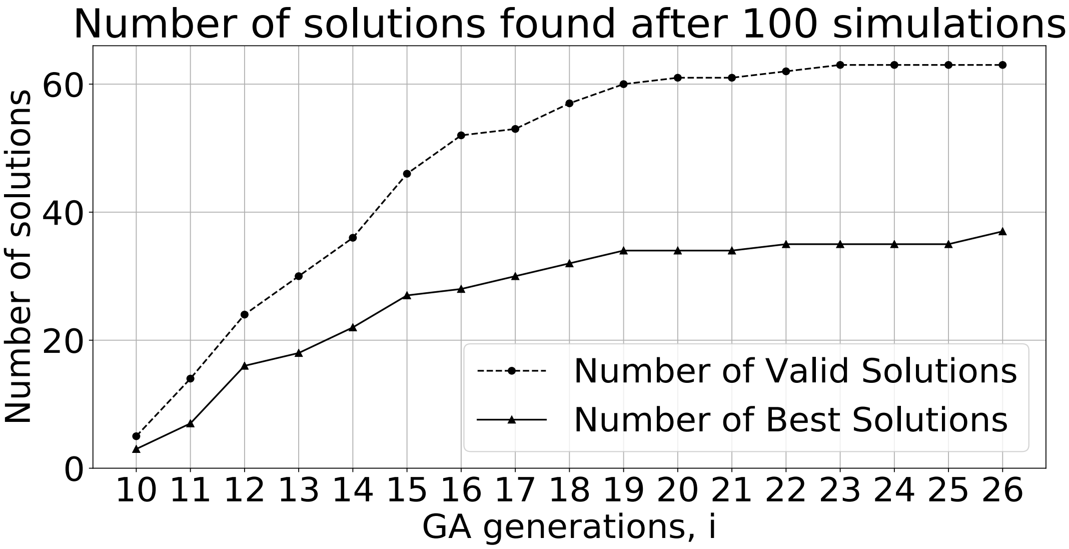

Figure S18: The number of valid and best solutions found by Hybrid Quantum Algorithm with Genetic Optimization (HQAGO) with 5 fixed qubits (representing a classic GA) after running the algorithm 100 times. The best outcome, in terms of the best and valid solutions, is achieved after 21 GA generations.

1.2 Graph coloring problem

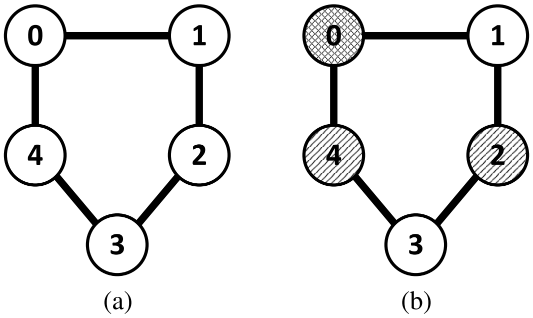

Figure S19: Panels a) and b) show the Erdős-Rényi graph generated with edge probability 0.7 and 5 nodes, and the solution that colors the graph.

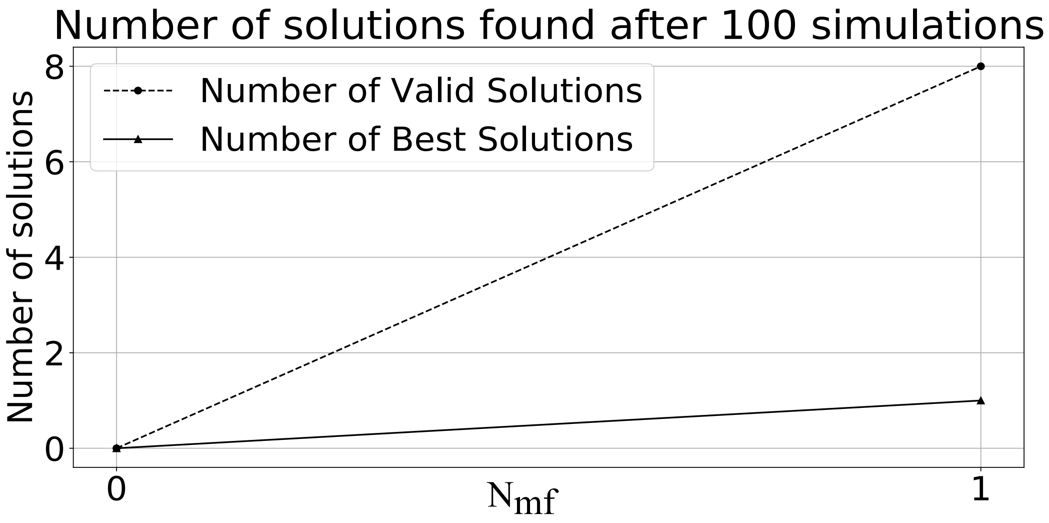

Figure S20: The number of valid and best solutions found by Hybrid Quantum Algorithm with Genetic Optimization (HQAGO) after running the algorithm 100. We simulate the algorithm with 1 fixed gene. The best outcome, in terms of the best and valid solutions, is achieved after 2 RQGA iterations.

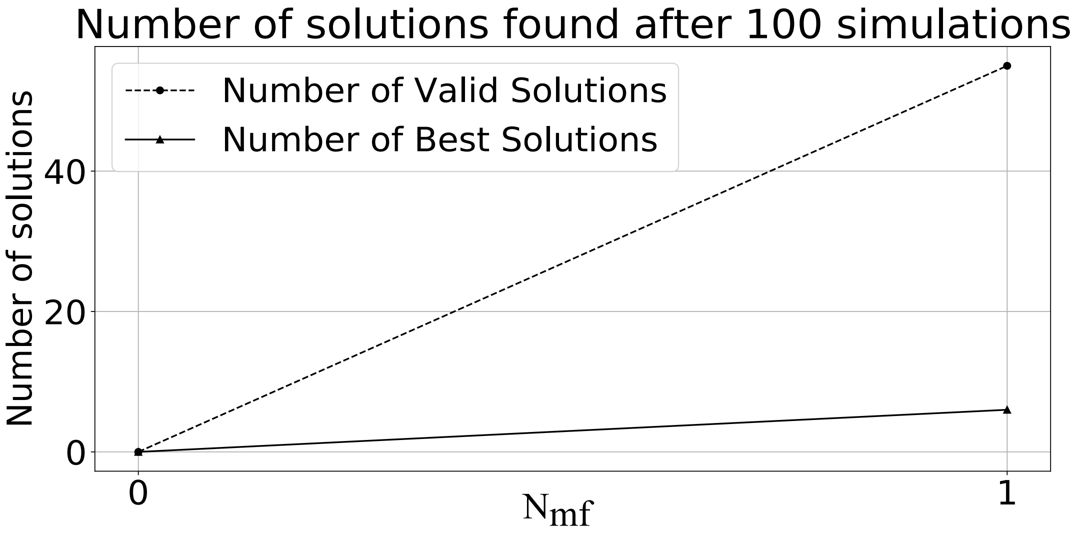

Figure S21: The number of valid and best solutions found by Hybrid Quantum Algorithm with Genetic Optimization (HQAGO) with 2 fixed individuals after running the algorithm 100 times. The best outcome, in terms of the best and valid solutions, is achieved after 2 RQGA iterations.

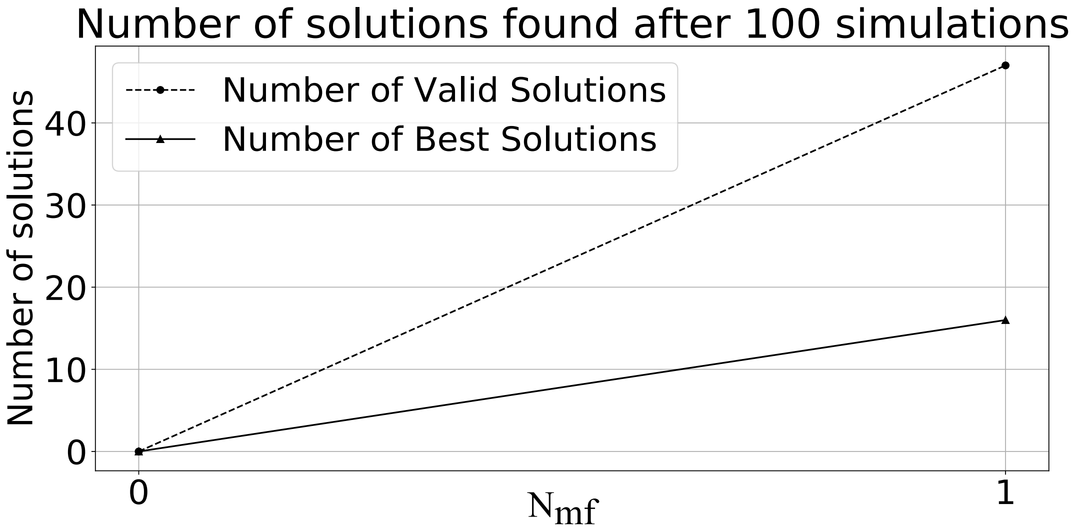

Figure S22: The number of valid and best solutions found by Hybrid Quantum Algorithm with Genetic Optimization (HQAGO) with 3 fixed individuals after running the algorithm 100 times. The best outcome, in terms of the best and valid solutions, is achieved after 2 RQGA iterations.

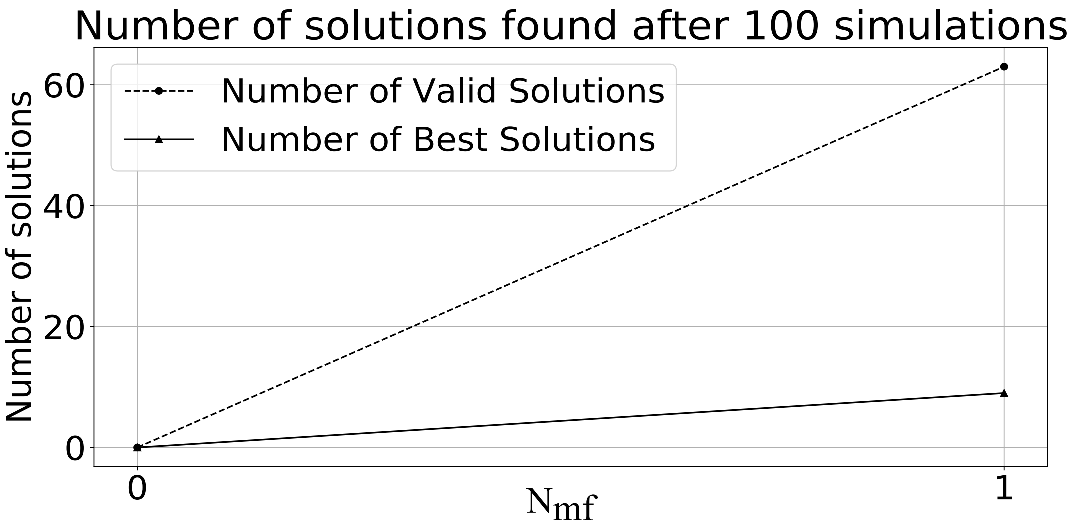

Figure S23: The number of valid and best solutions found by Hybrid Quantum Algorithm with Genetic Optimization (HQAGO) with 4 fixed individuals after running the algorithm 100 times. The best outcome, in terms of the best and valid solutions, is achieved after 2 RQGA iterations.

2 Knapsack problem example

This section presents an example of how the Hybrid Quantum Algorithm with Genetic Optimization is applied to solve an NP problem—the knapsack problem.

Problem: Considering a backpack with a maximum capacity of 10 kilograms and the following 4 items:

- $Item_0$ has 5 kg and a value of 20$
- $Item_1$ has 4 kg and a value of 10$
- $Item_2$ has 3 kg and a value of 30$
- $Item_3$ has 6 kg and a value of 40$

Solution We start by encoding the quantum chromosome on 4 qubits, where value 1 means that the item is present in the knapsack. Therefore, the chromosome will encode valid and non-valid individuals as superposed basis states, as presented in Table 1.

Items ($I_0I_1I_2I_3$)	Value [$]	Weight [kg]	Validity
0 0 0 0	0	0	Valid
0 0 0 1	40	6	Valid
0 0 1 0	30	4	Valid
0 0 1 1	70	9	Valid
0 1 0 0	10	4	Valid
0 1 0 1	50	10	Valid
0 1 1 0	40	7	Valid
0 1 1 1	80	13	Non-valid
1 0 0 0	20	5	Valid
1 0 0 1	60	11	Non-valid
1 0 1 0	0	8	Valid
1 0 1 1	90	14	Non-valid
1 1 0 0	30	9	Valid
1 1 0 1	70	15	Non-valid
1 1 1 0	60	12	Non-valid
1 1 1 1	100	18	Non-valid

Table 1: Chromosome binary configurations with their corresponding weight, value and validity flag.

We start by initializing a superposition of all individual-fitness register pairs as:

$$\begin{aligned} |\psi\rangle_1 &= \frac{1}{4}\sum_{u=0}^{15} |u\rangle_{ind} \otimes |0\rangle_{fit} \\ &= \frac{1}{4}\begin{pmatrix} & |0000\rangle & + & |0001\rangle \\ + & |0010\rangle & + & |0011\rangle \\ + & |0100\rangle & + & |0101\rangle \\ + & |0110\rangle & + & |0111\rangle \\ + & |1000\rangle & + & |1001\rangle \\ + & |1010\rangle & + & |1011\rangle \\ + & |1100\rangle & + & |1101\rangle \\ + & |1110\rangle & + & |1111\rangle \end{pmatrix} \otimes |000000000\rangle . \end{aligned} \tag{1}$$

Next, we apply the classical Genetic Algorithm (GA) to fix a subset of k-qubits, $0 \leq k \leq n$. In Equation (1) $|u\rangle \in S = \{0,1,2,\ldots,15\}$, and $u \in \mathbb{N}$ is binary-encoded ($u = b_0 b_1 b_2 b_3$ where $b_i \in \mathbb{B} = \{0,1\}$, $i = \overline{0,3}$). When we assign the classical binary values to $k = 2$ b_is then $|u\rangle \in S_k \subseteq S$; the cardinality of S_k is $|S_k| = 4$ elements. As such, the quantum state after applying the classical GA is:

$$\begin{aligned} |\psi\rangle_2 = GA\,|\psi_1\rangle &= \frac{1}{2}\sum_{u \in S_k} |u\rangle_{ind} \otimes |0\rangle_{fit} \\ &= \frac{1}{2}\begin{pmatrix} & |00①①\rangle & + & |01①①\rangle \\ + & |10①①\rangle & + & |11①①\rangle \end{pmatrix} \otimes |000000000\rangle, \end{aligned} \tag{2}$$

where the circled bits are fixed.

We apply the U_{fit} operator characterized by the fitness function $f_{fit} : \{0,1\}^n \to \{0,1\}^{m+1}$:

$$f_{fit}(x) = val(x) - (val_t + 1) \times (m(x) \nabla \cdot m_{max}) \tag{3}$$

as presented in (Udrescu et al., 2006). Therefore, the quantum state after applying the U_{fit} operator is:

$$|\psi\rangle_3 = U_{fit}\,|\psi\rangle_2 = \frac{1}{2}\sum_{u \in S_K} |u\rangle_{ind} \otimes |f_{fit}(u)\rangle_{fit} = \frac{1}{2}\begin{pmatrix} & |00①①\rangle & \otimes|100011110\rangle \\ + & |01①①\rangle & \otimes|011010111\rangle \\ + & |10①①\rangle & \otimes|011100001\rangle \\ + & |11①①\rangle & \otimes|011111111\rangle \end{pmatrix}. \tag{4}$$

We represent the fitness value in two's complement with the most significant bit–bit 9– dedicated to indicating the validity of the individual–0 indicates a non-valid individual, while 1 indicates a valid one. Therefore, the fitness values of the non-valid individuals are negative numbers.

In the next step, we apply the Oracle and Grover diffuser $(N-1)$ times, where $N = \mathcal{O}\left(\sqrt{2^{(n-k)}}\right)$ is the number of Grover iterations. Considering that the yielded value for *max* is 32, in Equations (5) we present the quantum state after subtracting the max value and applying phase shift. After applying the Oracle we obtain the quantum state shown in Equation (6).

$$|\psi\rangle_4^1 = |u\rangle_{ind} \otimes |f_{fit}(u)\rangle_{fit} \quad = \frac{1}{2}\begin{pmatrix} - & |00①①\rangle & \otimes|111111110\rangle \\ + & |01①①\rangle & \otimes|010110111\rangle \\ + & |10①①\rangle & \otimes|011000001\rangle \\ + & |11①①\rangle & \otimes|011011111\rangle \end{pmatrix} \tag{5}$$

$$\begin{aligned} |\psi\rangle_4 = \tilde{\mathbb{O}}_{max}\,|\psi\rangle_3 &= (-1)^{g(u)}\frac{1}{2}\sum_{u \in S_k} |u\rangle_{ind} \otimes |f_{fit}(u)\rangle_{fit} \\ &= \frac{1}{2}\begin{pmatrix} - & |00①①\rangle & \otimes|100011110\rangle \\ + & |01①①\rangle & \otimes|011010111\rangle \\ + & |10①①\rangle & \otimes|011100001\rangle \\ + & |11①①\rangle & \otimes|011111111\rangle \end{pmatrix}, \end{aligned} \tag{6}$$

where

$$g(u) = \begin{cases} 1 & if\,|f_{fit}(u)\rangle_{fit} \geqslant max \\ 0 & otherwise. \end{cases} \tag{7}$$

Next, we apply the diffuser operation over the fitness register from $|\psi\rangle_4$ and we get the state $|\psi\rangle_5$:

$$|\psi\rangle_5 = \mathbb{G}\,|\psi_4\rangle \quad = \frac{1}{2}\begin{pmatrix} - & \alpha_0\,|00\textcircled{1}\textcircled{1}\rangle & \otimes|100011110\rangle \\ + & \alpha_1\,|01\textcircled{1}\textcircled{1}\rangle & \otimes|011010111\rangle \\ + & \alpha_2\,|10\textcircled{1}\textcircled{1}\rangle & \otimes|011100001\rangle \\ + & \alpha_3\,|11\textcircled{1}\textcircled{1}\rangle & \otimes|011111111\rangle \end{pmatrix} \tag{8}$$

with amplitudes $\alpha_1, \alpha_2, \alpha_3 \approx 0$ and $|\alpha_1|^2 = 1$. If we measure the individual register of $|\psi\rangle_5$ we obtain, with a high probability, the following basis state: $|0011\rangle$. Therefore, we update the *max* value with the corresponding fitness value and repeat the above steps until the *max* value is no longer improved.

3 Code availability

All data needed to reproduce the results and evaluate the conclusions are in the paper. The design and implementation of HQAGO and the experiments are available on GitHub: `https://github.com/sebastianardelean/hqago`.

4 Acronyms

GA Genetic Algorithm. 1, 4, 7, 10, 14

HQAGO Hybrid Quantum Algorithm with Genetic Optimization. 1–12

RQGA Reduced Quantum Genetic Algorithm. 1–9, 11, 12

References

Udrescu, M., Prodan, L., and Vlăduţiu, M. (2006). Implementing quantum genetic algorithms: a solution based on grover's algorithm. In *Proceedings of the 3rd Conference on Computing Frontiers*, pages 71–82.
